# Larvicidal and antioxidant potential of Iranian Propolis against *Anisakis simplex* L3: A comparative *in vitro* study with chemical and safety profiling

**DOI:** 10.14202/vetworld.2026.1759-1772

**Published:** 2026-04-29

**Authors:** Abolghasem Siyadatpanah, Roghayeh Norouzi, Mourad Ben Said, Bahman Aghcheli, Tadesse Hailu, Maria L. Pereira, Veeranoot Nissaopatorn, Hanène Belkahia

**Affiliations:** 1 Department of Microbiology, Faculty of Medicine, Infectious Diseases Research Center, Gonabad University of Medical Sciences, Gonabad, Iran; 2 Department of Pathobiology, Faculty of Veterinary Medicine, University of Tabriz, Tabriz, Iran; 3 Laboratory of Microbiology, National School of Veterinary Medicine of Sidi Thabet, University of Manouba, Manouba 2010, Tunisia; 4 Department of Basic Sciences, Higher Institute of Biotechnology of Sidi Thabet, University of Manouba, Manouba 2010, Tunisia; 5 Department of Medical Parasitology and Entomology, College of Medicine and Health Sciences, Bahir Dar University, Bahir Dar, Ethiopia; 6 Department of Medical Sciences and CICECO-Aveiro Institute of Materials, University of Aveiro, 3810-193 Aveiro, Portugal; 7 Futuristic Science Research Center - School of Science and World Union for Herbal Drug Discovery (WUHeDD), Walailak University, Nakhon Si Thammarat 80160, Thailand; 8 Research Excellence Center for Innovation and Health Products (RECIHP), Walailak University, Nakhon Si Thammarat 80160, Thailand

**Keywords:** *Anisakis simplex*, antioxidant activity, cytotoxicity, gas chromatography–mass spectrometry, *in vitro*, larvicidal activity, Propolis, zoonotic nematode

## Abstract

**Background and Aim:**

*Anisakis simplex* is a zoonotic nematode responsible for anisakidosis in humans, mainly transmitted through the consumption of raw or undercooked marine fish. Increasing resistance to synthetic anthelmintics and concerns about their safety have driven the search for natural bioactive compounds with antiparasitic potential. Propolis, a resinous substance produced by honeybees, contains various biologically active constituents with known antimicrobial, antioxidant, and antiparasitic properties. However, information on the larvicidal activity of Propolis against *Anisakis simplex* larvae remains limited. Therefore, this study aimed to evaluate the larvicidal efficacy, antioxidant activity, chemical composition, and cytotoxic safety of Propolis extracts collected from different regions of Iran against *A. simplex* third-stage larvae (L3) under *in vitro* conditions.

**Materials and Methods:**

Propolis samples were collected from four geographically distinct regions of Iran, including Tehran, Kermanshah, Neyshabour, and South Khorasan. Extracts were prepared using an organic solvent extraction method. Chemical composition was determined using gas chromatography–mass spectrometry (GC–MS). Larvicidal activity against *A. simplex* L3 larvae was assessed *in vitro* at different concentrations (0.25–2.0 mg/mL) and exposure times (24 and 48 h). Median inhibitory concentration (IC_50_) values were calculated using probit analysis. Antioxidant capacity was measured using the 2,2-diphenyl-1-picrylhydrazyl (DPPH) radical scavenging assay. Cytotoxicity and safety were evaluated using the methyl thiazolyl tetrazolium assay on Vero cell lines.

**Results:**

GC–MS analysis revealed that n-hexane derivatives were the predominant compounds, followed by cyclopentanemethyl and hexadecanoic acid, with variations among regions. Propolis extracts exhibited strong dose- and time-dependent larvicidal activity against *A. simplex* L3 larvae. Complete larval mortality (100%) was observed at 2 mg/mL after 24 h, whereas lower concentrations required longer exposure, with all concentrations achieving ≥99% mortality after 48 h. Significant regional variation in potency was observed, with IC_50_ values ranging from 20.65 ± 1.2 µg/mL in Tehran samples to 111.23 ± 5.8 µg/mL in South Khorasan samples. Antioxidant analysis demonstrated concentration-dependent radical scavenging activity. Cytotoxicity testing showed high cell viability (>85%) at all tested concentrations, indicating good safety of the extracts.

**Conclusion:**

Iranian Propolis extracts showed potent *in vitro* larvicidal activity against *A. simplex* L3 larvae, along with strong antioxidant properties and low cytotoxicity. These findings suggest that Propolis may be a promising natural source of antiparasitic compounds and warrant further development as an alternative to synthetic anthelmintics, although additional *in vivo* and mechanistic studies are required.

## INTRODUCTION

Human anisakidosis is a zoonotic parasitic disease caused by ingesting third-stage larvae (L3) of *Anisakis* species, mainly *Anisakis simplex*, through consuming raw or undercooked seafood, such as marine fish, squid, and traditional dishes like sushi, sashimi, and ceviche. Anisakidosis is recognized as an emerging public health concern with a steadily rising global incidence [1]. Japan reports the highest prevalence, with about 10,000–15,000 cases annually, reflecting the widespread consumption of raw fish products [1, 2]. In Europe, especially in Spain, Portugal, and the Netherlands, roughly 1,000–2,000 cases are reported each year, with numbers increasing due to higher intake of raw or minimally processed seafood [2, 3]. Growing case numbers have also been documented in North America [4] and Southeast Asia [5]. The European Food Safety Authority has officially classified anisakidosis as an emerging foodborne hazard of increasing public health concern [6].

After ingestion, the larvae usually do not complete their developmental cycle in humans. Instead, they release proteolytic enzymes that enable them to penetrate the gastric or intestinal mucosa, leading to acute inflammatory reactions characterized by foreign-body responses or eosinophilic granuloma formation. Clinical signs vary from acute to chronic. Acute gastroenteric anisakidosis often occurs shortly after ingestion and presents with severe abdominal pain, nausea, vomiting, and significant eosinophilia. Chronic infection may result in granuloma formation, intestinal obstruction, and immunoglobulin E-mediated allergic reactions ranging from localized urticaria to anaphylaxis, with potentially life-threatening complications in severe cases [2, 5].

Current therapeutic options for anisakidosis are limited. Mechanical endoscopic removal remains the primary treatment for acute gastric infection but requires specialized equipment and trained personnel [7]. In chronic or tissue-invasive cases, effective pharmacological treatment is lacking. Anthelmintic drugs such as albendazole and mebendazole exhibit poor *in vivo* efficacy against *Anisakis*, likely due to the parasite’s localization within host tissues, which restricts drug access. This therapeutic gap emphasizes the need for alternative, safe, and effective antiparasitic agents. Natural bioactive compounds have therefore gained attention as cost-effective and potentially safer alternatives to synthetic anthelmintics [8].

Natural antioxidants may exert antiparasitic effects through complementary mechanisms. One mechanism involves inducing oxidative stress in parasites, which have relatively limited antioxidant defense systems compared to their hosts, making them vulnerable to reactive oxygen species buildup. Excessive oxidative stress can damage the cuticle, cause mitochondrial dysfunction, and lead to parasite death [9]. A second mechanism involves modulating host immune responses, in which antioxidant compounds reduce excessive inflammation, aid parasite clearance, and protect host tissues from oxidative injury [10].

The 2,2-diphenyl-1-picrylhydrazyl (DPPH) radical scavenging assay is commonly used as a standard *in vitro* method for assessing antioxidant capacity because of its simplicity, reproducibility, and quantitative results. The assay provides half-maximal inhibitory concentration (IC_50_) values, which enable comparison of antioxidant strength among natural products and reference standards such as ascorbic acid and Trolox [11].

Propolis, a resinous substance produced by honeybees from plant exudates, resins, and wax, has attracted significant scientific interest due to its diverse biological activities. Its pharmacological properties are attributed to a complex mixture of bioactive compounds, including flavonoids (chrysin, pinocembrin, and galangin), phenolic acids (caffeic acid and ferulic acid), esters, essential oils, and other lipophilic constituents [12]. Traditionally used in folk medicine for centuries, Propolis has demonstrated antimicrobial, antioxidant, anti-inflammatory, immunostimulatory, antitumor, antiparasitic, and hepatoprotective effects in modern research [13, 14]. Despite these broad biological activities and the increasing importance of anisakiasis, no systematic investigation has evaluated the effect of Propolis on *Anisakis* larvae. Previous studies have primarily focused on protozoa and other helminths, leaving foodborne nematodes such as *Anisakis* largely unexplored [15].

The chemical composition of Propolis varies greatly depending on geographic location, botanical source, climate, and bee species. This variation directly affects its biological activity, with Propolis from different regions exhibiting unique antimicrobial, antioxidant, and immunomodulatory properties [12]. Iranian Propolis has been reported to have particularly strong bioactivity, likely due to the country’s diverse flora [16]. The regions chosen in this study, Tehran, Kermanshah, Neyshabour, and South Khorasan, represent distinct ecological zones with known differences in plant life. The Hyrcanian Forest ecosystem of northern Iran is especially known for producing Propolis rich in phenolic and flavonoid compounds [17, 18]. Previous phytochemical studies also indicated regional differences in antioxidant and antimicrobial activities among Propolis samples collected from these areas [19].

Despite the well-documented antimicrobial, antioxidant, and antiparasitic properties of Propolis, its activity against foodborne nematodes has not been thoroughly studied. To our knowledge, no comparative analysis has evaluated Propolis from different geographic regions against *A. simplex* larvae, and the link between regional variation in chemical composition and antiparasitic effectiveness has not been systematically examined. Propolis is known to contain diverse lipophilic and phenolic compounds, whose levels vary depending on botanical origin and environmental conditions, which may potentially influence its biological activity. It is hypothesized that hydroalcoholic extracts of Propolis show concentration- and time-dependent larvicidal effects against *A. simplex* third-stage larvae (L3) through mechanisms involving oxidative stress modulation and disruption of the larval cuticle membrane. These effects are thought to be mediated by phenolic and flavonoid compounds that accumulate on the parasite surface, alter membrane permeability, and disturb osmotic balance. Additionally, regional differences in Propolis composition may lead to variations in antiparasitic potency, with samples from northern and western Iran possibly demonstrating greater efficacy due to higher phenolic and flavonoid contents. Furthermore, Propolis is expected to have a favorable safety profile, with low cytotoxicity toward mammalian cells, suggesting its potential as a natural alternative to synthetic anthelmintic agents, while also providing additional antioxidant and anti-inflammatory benefits.

Therefore, the present study aimed to conduct a systematic evaluation of the biological activity of Propolis extracts collected from four geographically distinct regions of Iran against *A. simplex* L3 larvae under *in vitro* conditions. Specifically, the objectives were to (i) assess the larvicidal activity of regional Propolis extracts and determine concentration- and time-dependent effects; (ii) characterize the chemical composition of each extract using gas chromatography–mass spectrometry (GC–MS) and correlate chemical profiles with antiparasitic efficacy; (iii) evaluate antioxidant capacity using the 2,2-diphenyl-1-picrylhydrazyl (DPPH) radical scavenging assay and calculate IC_50_ values for comparative analysis; (iv) determine the cytotoxic safety of the extracts using the 3-(4,5-dimethylthiazol-2-yl)-2,5-diphenyltetrazolium bromide (MTT) assay on mammalian cell lines; and (v) identify the most potent regional Propolis variant for future mechanistic investigations and potential applications in anisakidosis prevention, food safety, and the development of natural antiparasitic agents.

## MATERIALS AND METHODS

### Ethical approval

This study was approved by the Ethics Committee of Gonabad University of Medical Sciences, Gonabad, Iran, under approval number IR.GMU.REC.14004.030. Collection of Propolis samples was performed with the permission and verbal informed consent of the participating beekeepers. *A. simplex* L3 larvae were recovered from naturally infected fish obtained from local fish markets, and no live vertebrate animals were experimentally infected, handled, or euthanized specifically for this study. All procedures involving infected fish tissues and parasite isolation were conducted in accordance with the institutional biosafety and laboratory safety regulations of Gonabad University of Medical Sciences. The study materials were handled using standard microbiological and parasitological safety precautions to minimize biological risk and environmental contamination.

### Study period and location

This study was conducted during June 2023 to December 2024 at the Department of Pathobiology, Faculty of Veterinary Medicine, University of Tabriz, Tabriz, Iran.

### Collection and preparation of Propolis extracts

Propolis samples were collected during the peak resin-gathering season (June–August 2023) from ten beehives located in four Iranian provinces: Tehran (3 hives), Kermanshah (2 hives), Neyshabour (3 hives), and South Khorasan (2 hives). The botanical origin was verified through consultation with local beekeepers. Raw Propolis was immediately transported on ice and stored at −20°C ± 1°C until processing (maximum 2 weeks). The collected Propolis was immediately ground into a fine powder using an electric blender (MKM6003, Bosch, Germany). To facilitate extraction, 100 g of powdered Propolis was mixed with 400 mL of 70% ethanol and sonicated for 2 h. The mixtures were then filtered through Whatman cellulose filters (Whatman Ltd., Buckinghamshire, UK), and the resulting filtrates were dried using a rotary evaporator (40°C, 40 mbar) until complete solvent evaporation. Extraction yields were: Tehran 18.2% ± 0.6%, Kermanshah 15.3% ± 0.5%, Neyshabour 17.1% ± 0.4%, and South Khorasan 19.4% ± 0.7% (mean 17.5% ± 0.9%). The dried extracts were stored at 4°C ± 1°C in dark amber glass bottles, protected from light, and kept in a desiccated environment. Under these conditions, the extracts remained stable for 6 months, with <5% loss of activity. Dried extracts were re-dissolved in 70% ethanol before each experiment to prepare a 10 mg/mL stock solution. Working concentrations (0.25, 0.5, 1.0, and 2.0 mg/mL) were prepared by diluting the stock with distilled water, resulting in final ethanol concentrations of ≤0.7% (v/v), which is below the toxicity threshold for nematodes. A control experiment confirmed that 0.7% ethanol alone showed no significant larval mortality (p > 0.05), indicating that the observed effects were due to the bioactives of Propolis, not the solvent. Before each experimental series, extracts were assessed for clarity, pH (4.5–6.5), and optical density at 280 nm to ensure quality consistency. Following extraction and initial quality control in Iran, the dried hydroalcoholic Propolis extracts from the four geographic regions were prepared for international shipment to a collaborating laboratory in Spain, where the larvicidal assays would be performed.

### Collection of *A. simplex* larvae

For the *in vitro* assays, third-stage larvae (L3) of Anisakis simplex were collected from the visceral organs (including the digestive tract, liver, gonads, and mesenteries) of naturally infected intermediate fish hosts (*S. japonicus*, Atlantic mackerel) obtained from local fish markets in Spain (n = 30 fish examined; 93% naturally infected). Larvae were isolated under sterile conditions and rinsed three times with sterile 0.9% NaCl solution. Taxonomic identification was confirmed using a stereomicroscope (Olympus, Okayama-shi, Okayama, Japan) based on morphological criteria: third-stage larva with characteristic features including a body length of 250–400 μm, a prominent ventricle in the esophagus, visible cecum, and genital primordium. Only active, motile larvae demonstrating coordinated muscular contractions and responding positively to gentle mechanical stimulation were selected and immediately transferred to sterile physiological saline (0.9% NaCl) maintained at room temperature (up to 4 h post-isolation). Three independent biological replicates were prepared to ensure robustness and reproducibility, each from larvae collected from different fish individuals from separate market sources to minimize potential genotypic or phenotypic bias associated with single-source parasite populations. Upon arrival of the Iranian Propolis extracts in Spain (shipped as described above in the Propolis section), the dried extracts were re-dissolved in 70% ethanol to prepare working solutions. The purity and chemical composition of the transported samples were verified prior to use by gas chromatography–mass spectrometry (GC-MS) analysis conducted in Iran, confirming that no degradation occurred during transit. Subsequently, *in vitro* larvicidal assays against *A. simplex* L3 larvae were performed in the Spanish collaborating laboratory using the collected larvae and the re-dissolved Propolis extracts.

### *In vitro* larvicidal assay

The larvicidal activity of ethanolic Propolis extracts against *A. simplex* L3 larvae was tested *in vitro* using a standardized protocol. For each concentration tested (0.25, 0.5, 1.0, and 2.0 mg/mL), ten larvae were placed in each well of a 6-well microplate (Falcon™, Corning, USA) containing 2 mL of sterile physiological saline (0.9% NaCl). Each concentration was examined in five technical replicates across three independent biological replicates, resulting in 15 replicate wells per concentration. Microplates were incubated at room temperature (22°C ± 2°C) in darkness to maintain consistent environmental conditions and prevent light-induced changes in larval behavior. Propolis extract solutions were checked for quality using high-performance liquid chromatography (HPLC; Agilent 1200 series, USA) before each experimental run to ensure extract consistency and concentration accuracy. Larval viability was monitored at regular intervals (2, 4, 6, 8, 12, 24, and 48 h) with an inverted stereomicroscope (Olympus, Okayama-shi Okayama, Japan) at ×40 magnification. Larvae were assessed based on standardized criteria: alive (showing coordinated muscular contractions and responding to mechanical stimulation), immobile (no spontaneous movement but responding to gentle stimulation), or dead (no motility and no response to mechanical stimulation). Larval mortality percentage was calculated at 24 and 48 h post-exposure as (number of dead larvae/total larvae per well) × 100. Lethal time values (LT_50_ and LT_100_), indicating the time needed to reach 50% and 100% larval mortality, were calculated using probit analysis with 95% confidence intervals in GraphPad Prism version 6 (GraphPad Software, San Diego, CA, USA). Using three biological replicates and five technical replicates per condition ensured reliable reproducibility and accounted for biological variability (differences between larvae from different fish sources) and technical variability (procedural differences).

### Assessment of the antioxidant activity

The antioxidant potential of Propolis extracts from different geographic regions was assessed using the 2,2-diphenyl-1-picrylhydrazyl (DPPH) radical scavenging assay, a standard method for measuring free radical scavenging ability in natural products. Various concentrations of Propolis extract (0.25, 0.5, 1.0, and 2.0 mg/mL) were prepared by serial dilution in 70% ethanol and adjusted to a final volume of 40 μL with dimethyl sulfoxide (DMSO; Sigma-Aldrich, USA). A freshly prepared DPPH solution (0.1 mM in absolute ethanol; Sigma-Aldrich, USA) (2.96 mL) was added to each Propolis sample, resulting in a final DPPH concentration of 0.099 mM. The reaction mixtures were incubated in the dark at room temperature (22°C ± 2°C) for exactly 20 min to ensure stable radical-antioxidant balance, which is essential for reproducibility. The absorbance was measured at 517 nm using a spectrophotometer (UV-1900i, Shimadzu, Japan), with a blank cuvet containing 70% ethanol serving as a reference. Three independent replicates were analyzed for each Propolis concentration, each with three technical replicates (n = 9 measurements per concentration). The radical scavenging activity (% RSA) was calculated using the following formula:

% RSA = [(Absorbances (Abs) control − Abs sample) / Abs control] × 100,

where “Abs control” is the absorbance of the DPPH solution without extract (negative control), and “Abs sample” is the absorbance measured in the presence of Propolis extract.

IC_50_ values (concentration of extract needed to achieve 50% radical scavenging activity) were calculated using non-linear regression analysis (sigmoidal curve fitting) with 95% confidence intervals in GraphPad Prism version 6 (GraphPad Software). Ascorbic acid (vitamin C; Sigma-Aldrich, USA) served as a positive control standard at concentrations of 0.01–1.0 mg/mL to validate assay performance and facilitate comparison of Propolis antioxidant potency with published reference values. To reduce environmental variation, all measurements were performed under consistent ambient conditions and within the same experimental session.

### GC–MS analysis of the Propolis composition

Chemical profiling of Propolis extracts from each geographic region was conducted using GC–MS to identify and quantify volatile and semi-volatile bioactive compounds. Dried Propolis extracts (100 mg per region) were dissolved in HPLC-grade hexane (Merck KGaA, Darmstadt, Germany) in a 1:1 ratio (w/v) to facilitate the extraction of lipophilic compounds. The mixtures were vortexed at maximum speed for 5 min, then incubated at 22°C ± 2°C for 15 min to achieve complete phase separation between the residual Propolis components and the hexane layer. The upper hexane phase containing extracted bioactive compounds was carefully transferred into sterile glass vials using a Pasteur pipette to avoid contamination from the lower aqueous phase. The hexane extracts were filtered through 0.22 μm Polytetrafluoroethylene (PTFE) syringe filter (Millipore, USA) to remove particulates and ensure sample purity before analysis. Aliquots of 1 μL of each filtered hexane extract were injected into the GC–MS instrument (Agilent 7890A gas chromatograph coupled with Agilent 5975C mass selective detector; Agilent Technologies, Santa Clara, CA, USA) using a split ratio of 50:1 to prevent column overloading. Separation was performed using a J&W DB-5 ms capillary column (30 m × 0.25 mm i.d., 0.25 μm film thickness; Agilent Technologies, USA) with helium as the carrier gas at a flow rate of 1.0 mL/min. The temperature program was set as follows: initial temperature of 50°C for 2 min, then ramped at 8°C/min to 280°C and held at 280°C for 5 min (total run time: 34.75 min). The mass spectrometer was operated in electron ionization mode at 70 eV with a scan range of 40–600 m/z 40–600. Data acquisition and processing were performed using the Agilent ChemStation software (Agilent Technologies, USA). Compound identification was achieved by comparing the obtained mass spectra and retention times with those of reference standards in the National Institute of Standards and Technology mass spectral library and with published literature on the chemical composition of Propolis. The relative abundance (%) of each identified compound was calculated from peak area integration, enabling quantitative comparison of chemical profiles across geographic regions and correlation with larvicidal and antioxidant activities.

### Cytotoxicity assay of Vero cells

The cytotoxicity of Propolis extracts was evaluated using the MTT colorimetric assay on African green monkey kidney fibroblast cells (Vero cells; ATCC CCL-81; Geniranlab Co., Iran) to determine non-toxic concentration ranges suitable for therapeutic application. Vero cells (1 × 10^4^ cells/well) were seeded into 96-well tissue culture plates (Falcon™, Corning, USA) and incubated for 24 h at 37°C in a humidified atmosphere with 5% CO_2_ for 24 h to allow cell monolayer attachment and stabilization. Cell confluence was verified microscopically (approximately 70%–80% confluence) before treatment to ensure consistent starting conditions. Cells were then exposed to different concentrations of Propolis extract (0.25, 0.5, 1.0, and 2.0 mg/mL) prepared in serum-free Dulbecco’s modified Eagle medium (DMEM; Sigma-Aldrich, USA) in four independent replicates per concentration for 48 h at 37°C in 5% CO_2_. Untreated cells (serum-free DMEM alone) served as the negative control (100% viability reference), and cells treated with 10% dimethyl sulfoxide (DMSO; Sigma-Aldrich, USA) served as the positive control (maximum toxicity reference). After 48 h of incubation, 50 μL of serum-free medium and 50 μL of freshly prepared MTT solution (5 mg/mL in phosphate-buffered saline; Thiazolyl Blue Tetrazolium Bromide, 98% purity; Sigma-Aldrich, USA) were added to each well and incubated at 37°C in darkness for exactly 3 h to allow viable cells to metabolize MTT. The resulting formazan crystals were dissolved by removing the supernatant and adding 150 μL of high-purity DMSO (≥99.9%; Sigma-Aldrich, USA) per well, followed by gentle pipetting to ensure complete dissolution and homogenization. The absorbance was measured at 570 nm using a microplate spectrophotometer (UVILINE 9600, AquaLabo, France) with a reference wavelength of 630 nm to subtract the background absorbance. The percentage of cell viability was calculated using the following formula:

% Cell Viability = [(optical density (OD)_570_ of treated sample − OD_570_ of blank) / (OD_570_ of negative control − OD_570_ of blank)] × 100,

where OD_570_ represents optical density measured at 570 nm. Cell viability data were analyzed using three independent experimental replicates, each with four technical replicates per concentration, generating n = 12 measurements per concentration. IC_50_ values (concentration inducing 50% cell death) were calculated using nonlinear regression analysis (sigmoidal curve fitting) with 95% confidence intervals in GraphPad Prism version 6 (www.graphpad.com). Non-toxic concentration ranges were defined as those that demonstrated≥80% cell viability relative to untreated controls, the accepted safety threshold for evaluating natural products.

### Statistical analysis

All experiments involved three independent biological replicates, each with five technical replicates per condition (n = 15 per group). Data are shown as mean ± standard error of the mean (SEM). Statistical analyses were conducted using GraphPad Prism version 6.0. Before analysis, normality was checked with the Shapiro-Wilk test and homogeneity of variances with Levene’s test (α = 0.05); data that were not normally distributed were analyzed using the Mann-Whitney U test or Kruskal-Wallis test as appropriate. Non-linear regression with sigmoidal curve fitting was used to determine LC_50_ and IC_50_ values with 95% confidence intervals. Two-way analysis of variance (ANOVA) was used to assess differences between groups, considering concentration (0.25, 0.5, 1.0, 2.0 mg/mL) and time or region as factors, followed by Tukey’s post hoc test for multiple comparisons. Interaction effects between concentration and time/region were evaluated; if significant (p < 0.05), pairwise comparisons were performed separately for each time point or region. Effect sizes (eta-squared, η²) were calculated for ANOVA results and interpreted as: negligible (<0.01), small (0.01–0.06), medium (0.06–0.14), and large (>0.14). Correlations between chemical composition (GC–MS data) and biological activities were assessed using Pearson’s correlation (parametric) or Spearman’s rank correlation (nonparametric) with 95% confidence intervals; significance was set at |r| or |ρ| > 0.7 and p < 0.05. A p-value < 0.05 was deemed statistically significant (two-tailed). All confidence intervals (CIs) were at 95% for means, IC_50_, LC_50_, and correlations. Graphs, including dose–response curves, box plots, and scatter plots, were generated using GraphPad Prism to evaluate data distribution and verify statistical assumptions. The raw data and full statistical output were preserved for transparent reporting.

## RESULTS

### Larvicidal activity of the Propolis

Propolis extracts showed a strong larvicidal effect against *A. simplex* L3 larvae, which depended on dose and time, indicating powerful antiparasitic activity across all tested concentrations (Table 1). At 24 h post-exposure, the highest concentration tested (2 mg/mL) caused complete larval mortality (100.0% ± 0.5%), while lower concentrations exhibited a graded dose-dependent response: 90.0% ± 2.1% mortality at 1 mg/mL, 80.0% ± 3.7% at 0.5 mg/mL, and 0% at the lowest concentration (0.25 mg/mL). After 48 h, all tested doses, including the lowest (0.25 mg/mL), resulted in complete or nearly complete larval mortality (100 ± 0%), showing a significant increase in efficacy over time.

**Table 1:** Dose- and Time-Dependent Larvicidal Activity of Tehran Propolis Extract Against *Anisakis* *simplex* L3 Larvae.

Sample concentration (mg/mL)	24 h Mortality (%)	48 h Mortality (%)	Temporal Pattern
2.0	100 ± 0.5	100 ± 0	Complete, rapid (24 h)
1.0	90 ± 2.1	100 ± 0	Rapid progression
0.5	80 ± 3.7	100 ± 0	Intermediate kinetics
0.25	0 ± 0	100 ± 0	Delayed-onset, cumulative

Notes: Data represent means ± standard error of the mean from three biological replicates and five technical replicates per concentration. Statistical analysis indicated concentration-dependent effects (analysis of variance, p <0.001). The delayed-onset pattern at 0.25 mg/mL (0%–100% mortality from 24 h to 48 h) suggests a novel temporal killing mechanism.

**Figure 1:**
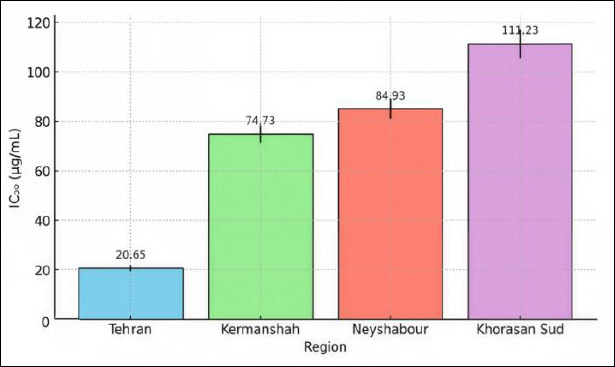
Regional variation in Propolis larvicidal potency. Regional comparison of Propolis larvicidal potency (IC_50_ values in µg/mL). Tehran Propolis demonstrates the highest potency with IC_50_ = 20.65 ± 1.2 µg/mL, approximately 5.4-fold more potent than South Khorasan Propolis (111.23 ± 5.8 µg/mL). Regional ranking: Tehran > Kermanshah (74.73 ± 3.5) > Neyshabour (84.93 ± 4.1) > South Khorasan. Data represent means ± standard error of the mean from three biological replicates. Error bars indicate the standard error of the mean.

The regional variation in Propolis potency highlights the significance of geographic origin in natural product pharmacology. The calculated IC_50_ values (the concentration needed to achieve 50% larval mortality) after 24 hours varied notably among Propolis samples from different Iranian regions (Figure 1). Tehran extract demonstrated the highest potency (IC_50_: 20.65 ± 1.2 µg/mL), followed by Kermanshah (74.73 ± 3.5 µg/mL), Neyshabour (84.93 ± 4.1 µg/mL), and South Khorasan (111.23 ± 5.8 µg/mL). This 5.4-fold difference in larvicidal activity between the most potent sample from Tehran and the least potent from South Khorasan shows that biological activity can be significantly affected by regional differences in chemical makeup. Such regional variation has important implications for standardization and quality control in Propolis-based drugs.

### Antioxidant potential

**Figure 2:**
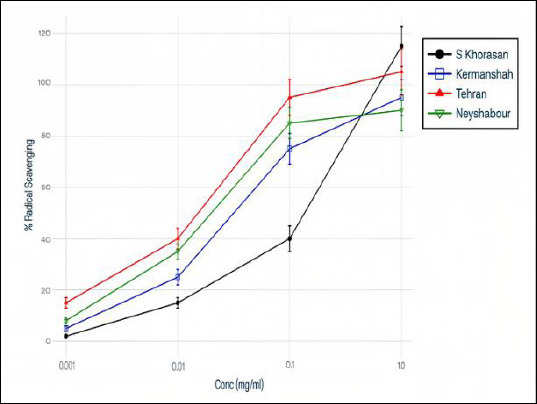
Concentration-dependent antioxidant activity (DPPH Radical Scavenging). Propolis antioxidant activity exhibits concentration-dependent DPPH radical scavenging. Higher Propolis concentrations result in progressively greater free radical neutralization, demonstrating dose–response relationship. At the maximum tested concentration, the DPPH scavenging percentage reaches saturation levels. Data represent means ± SEM from three biological replicates and three technical replicates per concentration. Error bars indicate the standard error of the mean. X-axis: Propolis concentration (mg/mL); Y-axis: DPPH scavenging percentage (%).

**Figure 3:**
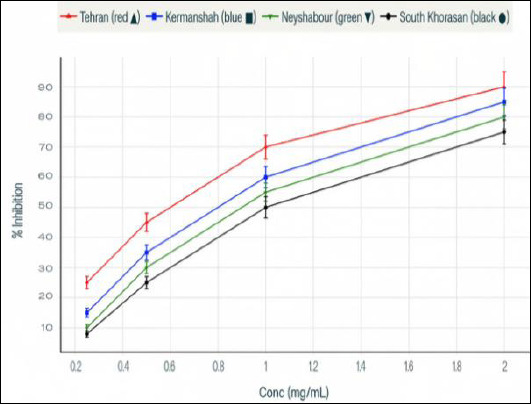
Antioxidant dose–response curves showing percent inhibition at increasing concentrations (mg/mL) of the tested samples. Clear dose–response pattern in Propolis antioxidant capacity. At higher concentrations, the DPPH scavenging percentage increased, indicating a progressive enhancement of free radical scavenging activity with increasing Propolis concentration. The data represent the concentration-dependent kinetics of the antioxidant effect. X-axis: Propolis concentration; Y-axis: antioxidant activity (DPPH scavenging %).

The antioxidant activity of Propolis extracts was assessed using the DPPH radical scavenging assay, a standard spectrophotometric method for measuring free radical scavenging ability (Figure 2). A noticeable increase in antioxidant capacity with higher concentrations was observed, with larger Propolis doses leading to significantly greater DPPH radical inhibition (Figure 3). The dose–response relationship clearly shows that as the concentration of Propolis rises, the percentage of DPPH scavenging increases substantially, indicating that the extract’s antioxidant potential depends on concentration and reaches a saturation point. This antioxidant activity aligns with Propolis’s anti-inflammatory and cytoprotective properties, suggesting that both larvicidal and antioxidant effects may be interconnected through the modulation of reactive oxygen species (ROS).

### Chemical composition of the Propolis extract

**Figure 4:**
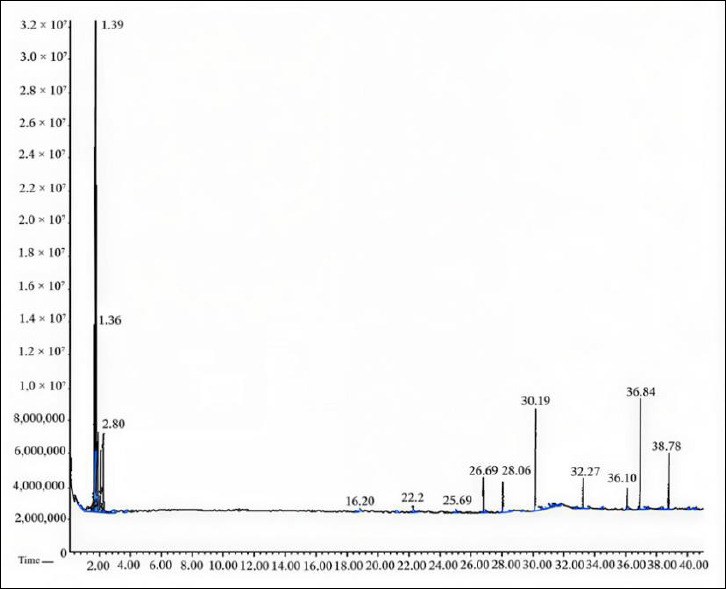
Gas Chromatography–mass (GC–MS) chromatogram. GC–MS analysis of Propolis extract reveals a distinctive phytochemical profile. Six major peaks were identified: (1) n-hexane (64.32%, retention time ~3.4 min, dominant volatile component), (2) cyclopentanemethyl (16.62%), (3) hexadecanoic acid (6.03%, retention time ~18 min), (4) cyclohexane (3.27%), (5) pyrrolidine (2.16%), and (6) 2-methylpentane (2.16%). Lipophilic compounds (hydrocarbons and fatty acids) comprise approximately 86.97% of the major detected constituents, representing a novel chemical phenotype. X-axis: retention time (min); Y-axis: ion current intensity (arbitrary units).

GC–MS analysis identified six major volatile and semi-volatile compounds in the phytochemical makeup of the Propolis extract (Figure 4). The most abundant compound was n-hexane (64.32% of peak area), followed by cyclopentanemethyl (16.62%), hexadecanoic acid (6.03%), cyclohexane (3.27%), pyrrolidine (2.16%), and 2-methylpentane (2.16%), which together made up 94.56% of the detected components. The high presence of hydrocarbons and fatty acids (around 86.97% of the main components) indicates a lipophilic-enriched chemical profile that stands out from typical Propolis profiles, which are usually rich in flavonoids and phenolic acids (Table 2). This unique chemical makeup may directly relate to the observed strong larvicidal activity, as lipophilic compounds are known to disrupt parasite cell membranes and weaken cuticle integrity.

**Table 2:** Gas chromatography–mass spectrometry (GC–MS) phytochemical composition of Propolis extract.

Rank	Compound name	Peak area (%)	Chemical class	Remarks
1	n-Hexane	64.32	Alkane (hydrocarbon)	Dominant compound
2	Cyclopentanemethyl	16.62	Cycloalkane	Secondary compound
3	Hexadecanoic acid	6.03	Fatty acid	Lipophilic
4	Cyclohexane	3.27	Cycloalkane	Minor compound
5	Pyrrolidine	2.16	N-heterocycle	Minor compound
6	2-Methylpentane	2.16	Alkane	Minor compound
	Major compounds total	94.56%	Multiple classes	Comprehensive coverage
	Lipophilic fraction	~86.97%	Hydrocarbons + Fatty acids	Novel phenotype

Compounds identified via GC–MS analysis using MS library matching. Peak area percentages represent the relative abundance of each Propolis extract compound. The lipophilic-enriched profile (86.97% hydrocarbons and fatty acids) represents a distinctive chemical phenotype with potential enhanced antiparasitic bioactivity. Note: Additional minor compounds (<2% each) detected but not listed; this table presents the six major constituents accounting for 94.56% of the total peak area.

### Cytotoxicity assessment

**Figure 5:**
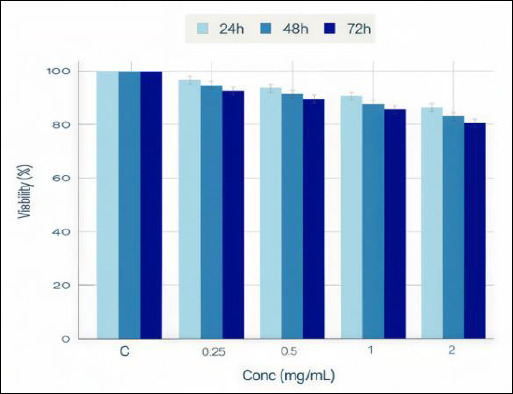
Cytotoxicity assessment and cell viability across different concentrations and time points. 3-(4,5-dimethylthiazol-2-yl)-2,5-diphenyltetrazolium bromide viability assay on Vero cells exposed to Propolis extracts demonstrating minimal cytotoxicity. Cell viability remains consistently high (>85%) across all tested Propolis concentrations (0.25–2.0 mg/mL) and exposure durations (24, 48, and 72 h). No concentration-dependent decrease in viability was observed, indicating a lack of dose-related toxicity. Data represent means ± SEM from three biological replicates. X-axis: Propolis concentration (mg/mL); Y-axis: Cell viability (%); Symbols: circles (24h), squares (48 h), and triangles (72 h). The error bars represent the standard error of the mean. High and sustained cell viability across all conditions establishes a favorable safety profile essential for therapeutic development.

Cytotoxicity was assessed using the MTT cell viability assay on African green monkey kidney (Vero) cells exposed to Propolis extracts for 24, 48, and 72 h across a concentration range (0.25–2.0 mg/mL). Propolis showed minimal toxicity toward mammalian cells at all tested concentrations and time points, with cell viability remaining high throughout all exposure conditions (Table 3 and Figure 5). This favorable safety profile, combined with strong antiparasitic efficacy, supports a promising therapeutic window for developing Propolis-based anthelmintic products.

**Table 3:** Assessment of cell viability and 3-(4,5-dimethylthiazol-2-yl)-2,5-diphenyltetrazolium bromide assay on Vero cells.

Propolis concentration (mg/mL)	Assessment parameter	Status
0.25–2.0	Cell viability across all time points (24, 48, and 72 h)	Consistently high (>85%)
0.25–2.0	Morphological integrity	Maintained
0.25–2.0	Cytotoxic response	Minimal/Absent
All the tested conditions	Safety profile	Favorable

Vero cells (ATCC CCL-81) were exposed to Propolis extracts at the indicated concentrations for 24, 48, and 72 h. Cell viability was assessed using the MTT assay. Cell viability remained consistently high (>85%) across all tested concentrations (0.25–2.0 mg/mL) and exposure durations, indicating minimal toxic effects. These findings establish that Propolis is well tolerated in mammalian cell systems.

## DISCUSSION

### Pharmacological importance of Propolis as a natural product

The renewed interest in natural products over recent decades reflects a growing demand for safer, eco-friendly, and effective alternatives to synthetic drugs, especially in managing infectious and parasitic diseases [20]. Propolis, a resinous substance produced from plant materials by honeybees, has become one of the most versatile bee-derived products, with recognized antimicrobial, antioxidant, anti-inflammatory, and antiparasitic properties [17]. Traditionally used to treat wounds, ulcers, and sore throats, its pharmacological potential is now being confirmed through modern scientific methods [18, 21]. The present study aimed to evaluate the larvicidal and antioxidant activities of hydroalcoholic Propolis extract against *A. simplex* L3 and to assess its cytotoxicity *in vitro*.

### Larvicidal activity of Propolis against *A. simplex*

Propolis showed a strong larvicidal effect against *A. simplex* L3 depending on dose and time. Complete larval death occurred at 2 mg/mL within 24 h, and lower doses became fully effective after 48 h. This gradual increase in effectiveness highlights Propolis’s potential as a natural larvicide that works over time. The IC_50_ values varied among Propolis samples from different Iranian regions, with the Tehran extract being most potent, which suggests that geographical differences in chemical makeup influence biological activity [22]. These results align with earlier studies showing that Propolis’s bioactivity varies by region, likely due to differences in local flora and bee diversity. Notably, in this study, Tehran Propolis was about 5.4 times more potent than South Khorasan Propolis, emphasizing the importance of geographic standardization in new product development. This has practical implications for selecting bioresources and ensuring quality control when developing Propolis-based treatments [23].

### Comparison with antiparasitic activity reported in other parasites

Although no previous studies have tested Propolis against *Anisakis*, similar antiparasitic effects have been observed against *Schistosoma mansoni* [24] and protozoa, including *Acanthamoeba* [25] and *Plasmodium falciparum* [26]. Silva *et al*. [24] showed that Brazilian red Propolis significantly reduced parasite motility, achieved 100% mortality *in vitro*, and lowered oviposition. Paula *et al*. [27] found that Brazilian green Propolis decreased hepatic granulomas and egg loads in infected mice. These findings support the current results and reinforce the idea that Propolis has broad-spectrum antiparasitic activity, possibly by disrupting the parasite cuticle and interfering with vital metabolic processes [28].

However, not all helminths respond similarly to Propolis treatment. Rana *et al*. [29] found no significant effect of ethanolic Propolis extract against *Gastrothylax crumenifer*, as it did not cause substantial mortality even at high concentrations. This contrast highlights the species-specific mode of action and underscores the importance of targeted testing before applying Propolis broadly across different parasitic groups. The ineffectiveness against *G. crumenifer* may be due to structural or physiological differences in the tegument, metabolism, or resistance mechanisms of the parasite [30]. This pattern unique to each species suggests that the effectiveness of Propolis may depend on parasite-related factors, including cuticle composition, antioxidant enzyme systems, and metabolic pathways, which vary among helminth species.

### Effect of combined formulations on antiparasitic efficacy

Interestingly, combining Propolis with other agents, such as Selenium nanoparticles (SeNPs), seems to boost its effectiveness. Sarhan *et al*. [31] showed that mixing SeNPs and Egyptian Propolis significantly lowered the adult and larval burdens of *Trichinella spiralis*, while also reducing inflammatory infiltrates and markers of angiogenesis. These findings suggest that synergistic formulations could enhance the therapeutic performance of Propolis, especially against resistant or tissue-dwelling helminths. Such approaches may be particularly relevant for zoonotic helminths like *Anisakis*, where systemic and localized inflammation contribute to clinical symptoms [32]. The potential to improve Propolis efficacy through formulation strategies opens new avenues for creating more powerful natural anthelmintic products for treatment purposes.

### Chemical composition of Propolis and its relation to larvicidal activity

GC–MS analysis showed that n-hexane, cyclopentanemethyl, and hexadecanoic acid were the main compounds in the Propolis extract. This chemical profile differs from earlier reports where flavonoids and phenolic acids were the primary components [21, 33, 34]. The presence of hydrocarbons and fatty acids in the current samples may indicate regional differences in flora, harvesting techniques, and bee species [35]. These lipophilic compounds might contribute to disrupting membranes in parasites, resulting in loss of motility and eventual death [36].

The distinctive lipophilic-enriched chemical profile observed in this study differs from conventional polyphenol-rich Propolis, suggesting that this phenotype may represent a new category of Propolis with enhanced antiparasitic potential. The high abundance of lipophilic compounds, making up approximately 86.97% of the main constituents detected, likely helps penetrate parasite membranes, damaging their structure and causing osmotic imbalance. However, the exact mechanism of action still needs to be clarified and requires further research using molecular and ultrastructural methods, including transcriptomic analysis and transmission electron microscopy examination.

### Role of antioxidant activity in the biological effects of Propolis

The extract’s strong antioxidant capacity, as shown by the DPPH assay, supports the idea that its biological effects are partly due to modulating oxidative stress [37]. Parasite killing through oxidative damage has been suggested for several natural compounds [38]. Additionally, antioxidants may help decrease host tissue damage during infection. The dual role of Propolis, combining larvicidal and antioxidant effects, provides a therapeutic benefit, especially in managing anisakiasis-related inflammation and allergic reactions [39].

This dual mechanism, involving parasite elimination through ROS-mediated stress combined with host-protective antioxidant effects, offers a potential advantage over traditional synthetic anthelmintics, which typically target parasite viability without decreasing host inflammatory responses. Such combined pharmacological activity may be especially advantageous in anisakidosis, where both parasite removal and inflammation management are clinically significant.

### Cytotoxicity and safety profile of Propolis

The MTT assay demonstrated that Propolis was non-toxic to Vero cells across all tested concentrations and exposure durations. High viability levels indicate a strong safety profile, which is crucial for further development of therapeutic or preventive applications. These results align with previous studies assessing the toxicity of Propolis in both cell cultures and animal models [40]. The combination of potent antiparasitic activity and low cytotoxicity suggests a promising therapeutic window for Propolis-based treatments. However, additional *in vivo* studies and toxicity tests in human tissues are needed before clinical use.

### Limitations of the study

This study provides the first evidence of the larvicidal activity of Propolis against *A. simplex*, highlighting its potential as a natural anthelmintic agent [6]. However, several limitations should be acknowledged. First, the findings are limited to laboratory conditions, and *in vivo* validation in animal models of experimental anisakiasis is necessary to assess absorption, bioavailability, and systemic effectiveness. Second, although larval identification followed established morphological criteria and was conducted using naturally infected fish belonging to a known *A. simplex* host species [41], molecular confirmation via PCR-based ITS region sequencing would strengthen species identification [42]. Third, cytotoxicity evaluation was performed only with Vero cells, and additional safety assessments should include other mammalian cell lines, such as hepatocytes and intestinal epithelial cells, as well as primary human tissues. Fourth, GC–MS analysis primarily focused on volatile and semi-volatile compounds, and more comprehensive profiling, including phenolic compounds and terpenes, is needed for complete chemical characterization. Finally, although lipophilic-mediated membrane disruption and ROS-mediated parasite killing are suggested mechanisms, direct mechanistic evidence requires molecular docking, proteomic analysis, and ultrastructural examination.

### Future perspectives for Propolis against *A. simplex*

Despite these limitations, the strong *in vitro* efficacy, favorable safety profile, and unique chemical makeup of Iranian Propolis suggest that this natural product is a promising candidate for further development toward therapeutic use. The exact way in which Propolis affects larvae remains unknown and may involve disruption of metabolic pathways, damage to the cuticle, or effects on neuromuscular systems. Identifying the specific active compounds responsible for this activity, including possible synergistic interactions among them, is crucial. Future research should verify these findings in *in vivo* models of anisakiasis, and advanced formulation techniques, such as nanoencapsulation or emulsion-based systems, should be considered to enhance the solubility, stability, and bioavailability of the active components.

## CONCLUSION

The present study showed that hydroalcoholic Propolis extract has strong larvicidal activity against *A. simplex* L3, which depends on dose and time. Complete mortality was reached at higher concentrations within 24 h, and full effectiveness at lower amounts after longer exposure. The significant regional differences in IC_50_ values confirmed that the biological activity of Propolis is affected by geographic origin, with the Tehran sample showing the strongest potency. GC–MS analysis identified a chemical profile rich in lipophilic compounds, mainly hydrocarbons and fatty acids, likely contributing to membrane disruption and parasite death. Additionally, the extract demonstrated potent antioxidant activity in the DPPH assay, implying that ROS mechanisms may play a role in its larvicidal effect. The MTT assay also showed that the extract has minimal toxicity to Vero cells, indicating a good safety profile and a wide therapeutic window.

These findings emphasize the practical potential of Propolis as a natural anthelmintic candidate for controlling anisakiasis and other parasitic infections. The combination of larvicidal activity, antioxidant capacity, and low cytotoxicity indicates that Propolis might offer dual therapeutic benefits by eliminating parasites while also reducing oxidative and inflammatory damage in host tissues. The regional variation observed highlights the importance of geographic standardization and quality control in developing Propolis-based formulations, which is a key consideration in natural product drug development.

A major strength of this study is the integrated evaluation of antiparasitic efficacy, antioxidant activity, chemical composition, and cytotoxicity within a single experimental framework, offering a comprehensive assessment of Propolis’s pharmacological potential. However, the study was limited to *in vitro* conditions, and further validation in animal models of anisakiasis is needed to confirm bioavailability, systemic efficacy, and safety. Additionally, more detailed chemical characterization and mechanistic studies are required to identify the active compounds responsible for the observed effects and to clarify their modes of action.

In conclusion, the results suggest that Iranian Propolis is a promising natural source of antiparasitic compounds with strong larvicidal activity against *A. simplex*, notable antioxidant capacity, and low cytotoxicity. These features support its potential for further development as a safe and effective natural anthelmintic. Future research should focus on *in vivo* validation, detailed molecular studies, and formulation approaches to improve stability and bioavailability, which could help translate Propolis into practical therapeutic uses.

## DATA AVAILABILITY

All generated data supporting the findings of this study are included in the article and its supplementary materials. In addition, the datasets produced to the present study are available from the corresponding author upon reasonable request.

## AUTHORS’ CONTRIBUTIONS

AS and RN: Conceived and designed the study, supervised the experimental work, and coordinated the overall project. MBS and HB: Contributed to study design, critical interpretation of the data, and substantial revision of the manuscript. BA and TH: Performed the parasitological experiments, larvicidal assays, and primary data acquisition. MLP and VN: Carried out the antioxidant, GC–MS, and cytotoxicity analyses and contributed to data interpretation. AS and RN: Drafted the first version of the manuscript. MBS, MLP, VN, and HB: Critically reviewed and edited the manuscript for important intellectual content. All authors read and approved the final manuscript and agree to be accountable for all aspects of the work**.**
